# Pharmacokinetic modeling strategies for dynamic hyperpolarized urea imaging

**DOI:** 10.1002/mrm.70117

**Published:** 2025-10-12

**Authors:** Keith A. Michel, Collin J. Harlan, Christopher M. Walker, Matthew E. Merritt, Yunyun Chen, Stephen Y. Lai, James A. Bankson

**Affiliations:** ^1^ Department of Imaging Physics The University of Texas MD Anderson Cancer Center Houston Texas USA; ^2^ Department of Radiology Houston Methodist Hospital Houston Texas USA; ^3^ Department of Biochemistry and Molecular Biology The University of Florida Gainesville Florida USA; ^4^ Department of Head and Neck Surgery The University of Texas MD Anderson Cancer Center Houston Texas USA

**Keywords:** hyperpolarized MR, pharmacokinetic modeling, urea

## Abstract

**Purpose:**

To evaluate pharmacokinetic modeling methods for quantification of tissue perfusion/permeability with hyperpolarized ^13^C urea.

**Methods:**

Three models for quantitative analysis of dynamic HP urea imaging data were proposed and evaluated in numerical simulations and a thyroid cancer mouse model. A multicompartment model resembling the extended Tofts model for DCE‐MRI (Model I) and two simplified models were used. The simplified models each eliminate a volume parameter representing vascular (Model II) or impermeable cellular space (Model III). Signal curves were generated from Model I, and models were fit to these synthetic data to quantify the effects of acquisition settings, bias in simplified models, and noise.

**Results:**

For Model I, reproducible and accurate results from snapshot imaging occurred at excitation angles of roughly 10 to 40 degrees, with wider ranges of good performance at longer TRs. Models II and III exhibited bias in estimation of the trans‐capillary transfer rate constant (*k*_*ve*_), with high sensitivity in *k*_*ve*_ fitting to variations in the volume parameter not explicitly included. At a peak SNR of 25, *k*_*ve*_ coefficients of variation were 14.6%, 5.53%, and 4.9% for Models I–III, respectively. When vascular input function (VIF) amplitude was jointly estimated, these coefficients of variation increased to 26.9%, 8.86%, and 25.4%. Individual pharmacokinetic parameters exhibit added bias with VIF amplitude fitting, but the *k*_*ve*_/*v*_*e*_ and *k*_*ve*_/*v*_*b*_ ratios are independent of VIF scaling and provide accurate results for Models I and III.

**Conclusion:**

Our results demonstrate the feasibility and relative performance of pharmacokinetic models for HP urea for quantification of tissue permeability and perfusion.

## 1 INTRODUCTION

Hyperpolarized (HP) MRI has demonstrated the capacity for real‐time assessment of metabolism and perfusion, enabling new functional imaging methods for cancer and many other diseases.^1^, ^2^ The most widely studied HP agent, [1–^13^C]pyruvate, can provide quantitative measurements of molecular flux through key metabolic pathways, but such measurements must account for the proportion of agent in the vasculature and variations in delivery rate to the site of metabolism.^3^ Several HP agents have been developed for perfusion imaging that permit measurement of vascular delivery effects.^4^, ^5^, ^6^ Among these, HP urea is notable as an endogenous molecule with a well‐established clinical safety profile^7^ and has been applied in preclinical investigations of renal^8^, ^9^ and cardiac^10^, ^11^ diseases. Recent work has demonstrated the clinical feasibility of simultaneous imaging of [1–^13^C]pyruvate and [^13^C,^15^N_2_]urea to measure both metabolism and perfusion,^12^ with results reported in prostate cancer.^13^.

Imaging of co‐polarized pyruvate and urea can detect tissue perfusion/metabolism mismatches within a single MRI exam, enabling evaluation of viable myocardium post‐infarction,^11^ identification of aggressive cancer,^14^, ^15^ and multiparametric assessment of tumor treatment response.^16^, ^17^ The extent to which delivery effects may bias or even dominate quantitative measurement of metabolism via HP MRI is currently under active investigation and may have profound impact on its clinical utility.^2^, ^18^ In this context, pharmacokinetic modeling of HP urea signal evolution can quantify vascular delivery effects in a way that is comparable with modeling methods for metabolic agents.^3^, ^19^ Similar to gadolinium‐chelate agents used in DCE‐MRI, HP urea in tissue with permeable vasculature extravasates from blood to the extravascular/extracellular space (EES).^6^ Various kinetic models analogous to those introduced for DCE‐MRI may therefore be suitable for quantitative analysis of dynamic urea images; however, no comparative study of the performance of different models for this purpose has been reported.

In this work, we evaluate pharmacokinetic modeling strategies for quantification of vascular perfusion and permeability from HP urea signal evolution. Model performance was studied through numerical simulations assessing the effects of acquisition settings, model complexity and noise level. The modeling strategies proposed were applied to dynamic HP urea imaging an orthotopic mouse model of anaplastic thyroid carcinoma to demonstrate their feasibility and to compare in vivo results with those derived from numerical simulations.

## 2 METHODS

Simulation experiments were conducted in MATLAB R2020b (The Mathworks, Natick, MA) to assess the performance of three pharmacokinetic modeling methods for quantitative analysis of HP urea signal evolution. Model performance was assessed in terms of the accuracy and reproducibility of the physiological parameters, known a priori, representing vessel permeability and physical volume fractions. In vivo images of HP urea in mice bearing orthotopic thyroid tumors were acquired and analyzed in a manner consistent with our numerical simulations as proof‐of‐concept for the proposed modeling methods. Animal experiments were performed with approval of our Institutional Animal Care and Use Committee.

### 2.1 Pharmacokinetic models

The primary kinetic model used in this work (Model I) is similar to the extended Tofts model for DCE‐MRI,^20^ and is defined by three physiological parameters corresponding to vascular perfusion and permeability (*k*_*ve*_), and volume fractions of blood (*v*_*b*_) and EES (*v*_*ee*_). In the absence of RF excitation, the change in concentration of labeled spins in the EES is determined by the vascular input function (VIF), T_1_ decay and HP agent extravasation/washout:

(1) $$\frac{\partial C_{e}\left(t\right)}{\partial t}=\frac{k_{\mathrm{ve}}}{v_{\mathrm{ee}}}C_{b}\left(t\right)-\left(\frac{k_{\mathrm{ve}}}{v_{\mathrm{ee}}}+\frac{1}{T_{1}}\right)C_{e}\left(t\right)=\frac{k_{\mathrm{ve}}}{v_{\mathrm{ee}}}C_{b}\left(t\right)-\alpha C_{e}\left(t\right)$$

where *C*_*b*_ *(t)* and *C*_*e*_ *(t)* represent the concentrations of labeled spins in the vascular space and EES, respectively, and *α* captures the effects of washout and T_1_ decay. The solution for this differential equation describes the concentration of labeled spins in the EES as a function of time, model parameters, and the vascular input function. The overall concentration of labeled spins in a given volume can be written as a weighted combination of spins from each physical compartment:

(2) $$C\left(t\right)=v_{b}C_{b}\left(t\right)+v_{\mathrm{ee}}C_{e}\left(t\right)+v_{c}C_{c}\left(t\right)$$

Recognizing that, over the timescale of an HP MRI observation, a negligible amount of HP urea is expected to cross into intracellular space in cells other than erythrocytes in most healthy tissues due to paucity of urea transport protein expression,^21^ the last term in Equation (2) can be omitted (*C*_*c*_ *(t)* = 0). Substituting the solution to Eq. (1) into Eq. (2) and recognizing that the observed signal is proportional to the concentration of labeled spins in a given volume, the governing equation for signal evolution in Model I can be written as:

(3) $$M_{z}\left(t\right)\propto v_{b}C_{b}\left(t\right)+v_{\mathrm{ee}}C_{e}\left(t_{0}\right)e^{-\alpha \left(t-t_{0}\right)}+k_{\mathrm{ve}}\int_{t_{0}}^{t}C_{b}\left(\tau \right)e^{-\alpha \left(t-\tau \right)}d\tau$$

Unlike DCE MRI modeling of gadolinium‐chelates, a hematocrit correction factor is not applied to the vascular volume fraction since erythrocytes are known to be highly permeable to urea.^22^

The remaining two models employed in this study can each be derived from Model I by elimination of a volume fraction parameter to reduce complexity and improve reproducibility of pharmacokinetic analyses. Model II resembles the Tofts model,^23^ with physiological parameters corresponding to vessel perfusion/permeability and extracellular volume fraction (*k*_*ve*_ and *v*_*ec*_). In this model, vascular signal is considered negligible and signal evolution follows the equations above, with *v*_*b*_ = 0:

(4) $$M_{z}\left(t\right)\propto v_{\mathrm{ec}}C_{e}\left(t_{0}\right)e^{-\alpha \left(t-t_{0}\right)}+k_{\mathrm{ve}}\int_{t_{0}}^{t}C_{b}\left(\tau \right)e^{-\alpha \left(t-\tau \right)}d\tau$$

Model III assumes the effect of the cellular volume fraction that is inaccessible to the HP perfusion agent is negligible, and accounts for physiological parameters *k*_*ve*_ and *v*_*b*_. This model is defined by the equations for Model I, with *v*_*ev*_ = 1‐*v*_*b*_:

(5) $$\begin{array}{cc} & M_{z}\left(t\right)\propto v_{b}C_{b}\left(t\right)+\left(1-v_{b}\right)C_{e}\left(t_{0}\right)e^{-\alpha \left(t-t_{0}\right)}\\ & \;+k_{\mathrm{ve}}\int_{t_{0}}^{t}C_{b}\left(\tau \right)e^{-\alpha \left(t-\tau \right)}d\tau \end{array}$$

**FIGURE 1:**
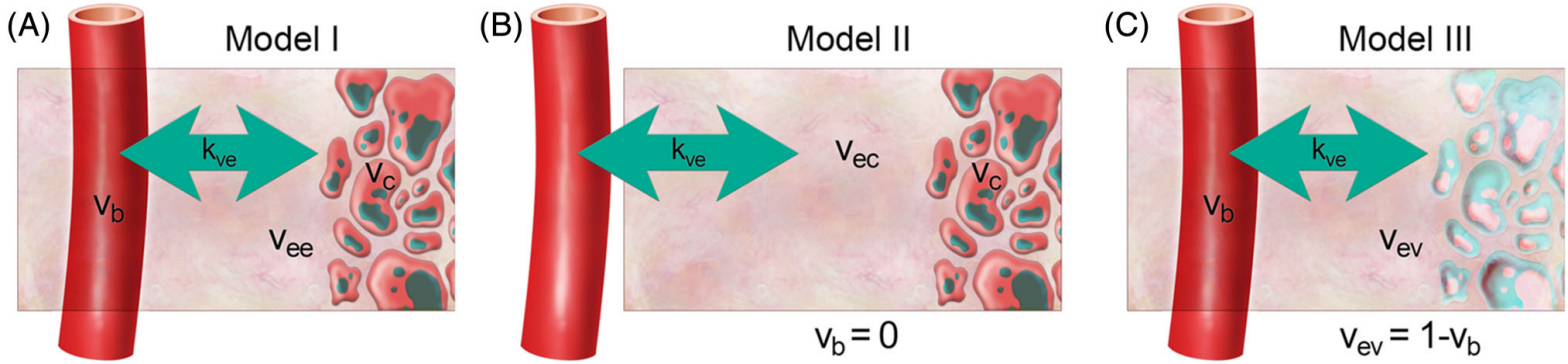
Three pharmacokinetic models of tissue permeability were considered in this work for the analysis of HP ^13^C urea images. (A) Model I is similar to the extended Tofts model for DCE, and is defined by three parameters representing the capillary perfusion and permeability (*k*_*ve*_), and the volume fractions of blood (*v*_*b*_) and extravascular/extracellular space (*v*_*ee*_). A cellular volume fraction (*v*_*c*_) inaccessible to the HP imaging agent is present in this model, but since all three volume fractions sum to unity it is not present as a separate parameter. (B) Model II resembles the Tofts model for DCE, with two parameters for perfusion/permeability (*k*_*ve*_) and extracellular volume fraction (*v*_*ec*_). Similar to Model I, an inaccessible cellular space is present in Model II but not as a separate parameter (*v*_*c*_ + *v*_*ec*_ = 1). (C) Model III includes only vascular (*v*_*b*_) and extravascular (*v*_*ev*_) volume fractions with bidirectional exchange (*k*_*ve*_) and the effects of an inaccessible cellular space are considered negligible (*v*_*c*_ = 0). This model can be defined by two parameters (*k*_*ve*_ and either volume fraction). When comparing tissue distribution volume fraction values between models (*v*_*ee*_ in Model I, *v*_*ec*_ in Model II, and *v*_*ev*_ in Model III), we use the generic parameter name *v*_*e*_.

Figure 1 shows graphical depictions of these three pharmacokinetic models. When comparing tissue distribution volume fractions between models (*v*_*ee*_, *v*_*ec*_ and *v*_*ev*_), we use the generic parameter name *v*_*e*_.

### 2.2 Numerical simulations

Synthetic signal curves were generated using Model I with parameters estimated from prior imaging experiments.^18^, ^24^ Unless otherwise stated, default reference values in generation of synthetic data were 0.02 s^−1^ for *k*_*ve*_, 9% for *v*_*b*_ and 30% for *v*_*ee*_. The T_1_ value for HP urea was assumed to be 20 s.^4^, ^25^ The VIF was modeled as a gamma‐variate function with shape terms matching those measured in small animal HP imaging exams.^24^, ^26^ Except for the T_1_ value for HP urea, these default parameters correspond to values measured for pharmacokinetic analysis of HP pyruvate in small animal models of solid tumors. Dynamic data were assumed to be acquired via a snapshot spoiled gradient‐echo sequence, with RF excitations modeled as instantaneous redistributions of longitudinal and transverse magnetization determined by a constant excitation angle.^27^, ^28^ The first excitation always coincided with the onset of the VIF (with negligible initial HP signal), and signal evolutions were simulated for 60‐s acquisitions. Except in evaluations of acquisition parameter effects, signal evolution was simulated for a 1‐s TR and 20‐degree excitation angle. Because we expect for blood within vessels in a given voxel to be largely replaced over the relatively long TR interval of an HP MRI measurement, excitation losses were not applied to the VIF.^29^

Nonlinear least‐squares fitting of models to simulated signal curves was performed with a trust‐region‐reflective conjugate‐gradient algorithm (using the MATLAB function lsqcurvefit).^30^ In simulations with noisy data, fitting was repeated 100 times with the addition of fresh Gaussian noise. To compare performance in the presence of noise consistently across differing physiological parameter values and acquisition settings, stated reference SNR values correspond to the variance of added Gaussian noise that produces this peak SNR for the default acquisition and model parameters as described in the previous paragraph.

The effects of acquisition settings on estimation of Model I parameters were evaluated through numerical simulations at a reference SNR of 25 with TRs ranging from 0.1 to 3 s and excitation angles from 1 to 90 degrees. Accuracy and reproducibility of parameter estimates were quantified as the mean error and coefficient of variation of the fit values.

The sensitivity of Models II and III to variations in the parameter values used to generate synthetic data with Model I was assessed in noise‐free simulations. Data were synthesized from Model I with parameter values varied through ranges of 0.002 to 0.5 s^−1^ for *k*_*ve*_, 2 to 50% for *v*_*b*_, and 2 to 50% for *v*_*ee*_. Individual Model I parameters were iterated through these ranges with the remaining two parameters held constant at default values. Parameter estimates from fitting Models II and III to these data demonstrate the relative bias and response of these simplified models to underlying changes in Model I.

The accuracy and reproducibility of parameter estimation for all three models was evaluated in noisy data with reference SNRs ranging from 10 to 50. These data were fit with an accurate VIF as a known, fixed set of parameters. Fitting was repeated with a VIF amplitude scaling factor as an additional fitted parameter. Differences in reproducibility of parameter estimates were assessed between fitting cases through comparison of coefficients of variation. Accuracy and bias in these results were evaluated by comparing parameter values to the known reference values. These results illustrate the relative performance for estimation of each physiological parameter between the three models, as well as the effects of joint estimation of VIF amplitude.

### 2.3 In vivo thyroid tumor imaging

Mouse imaging was performed on a 7T small animal MRI system with ParaVision 6.0.1 (Biospec 70/30 USR, Bruker Biospin MRI, Billerica, MA) using a 72‐mm inner diameter ^1^H/^13^C volume coil (Bruker Biospin MRI) for ^1^H imaging and for ^13^C RF excitation. Orthotopic tumors were produced by injection of U‐HTH83 anaplastic thyroid carcinoma cells into the thyroid glands of male athymic nude mice, as described previously.^31^ Mice (*N* = 4) were imaged in the supine position with a 20‐mm diameter surface coil (Rapid MRI International, Columbus, OH) for ^13^C signal reception placed anteriorly over the neck. Anesthesia was induced and maintained with 2–4% isoflurane delivered via nose cone.

Following ^1^H imaging to verify positioning of the mouse and surface coil, the signal from a concentrated ^13^C urea phantom placed along the animal's side was used to determine the ^13^C center frequency and transmit power. A solution of 100 mM HP [^13^C,^15^N]urea (Sigma Aldrich, St. Louis, MO) was produced using a HyperSense polarizer (Oxford Instruments, Abingdon, England) as described previously,^32^ and 200 μL was injected via a tail vein catheter. Snapshot ^13^C urea images were acquired dynamically with a TR of 1 s and TE of 11.2 ms for a single 1‐cm axial slice centered on both the thyroid tumor and ^13^C surface coil, starting prior to HP agent injection and continuing through the observable lifetime of the HP signal. Images were encoded using a single‐shot flyback EPI trajectory with a 4 × 4 cm FOV and 2.5 mm in‐plane resolution.^28^ An excitation angle of 20 degrees was used, consistent with the default acquisition parameters for numerical simulations. A ^1^H RARE sequence was acquired following the HP scan with matching FOV and slice position for use as an anatomical reference image.

Magnitude HP images were reconstructed in MATLAB and urea signal timecourses from a voxel near the base of the skull contralateral to the tumor and exhibiting a rapid, early increase in HP signal was used as the VIF shape for pharmacokinetic model fitting in each animal. Our three models were fit to HP signal timecourses from thyroid tumor voxels in a manner consistent with our numerical simulations, using the measured VIF shape and with the VIF scaling factor as a fitted parameter to account for variable receive coil sensitivity within the HP image.

## 3 RESULTS

### 3.1 Numerical simulations

**FIGURE 2:**
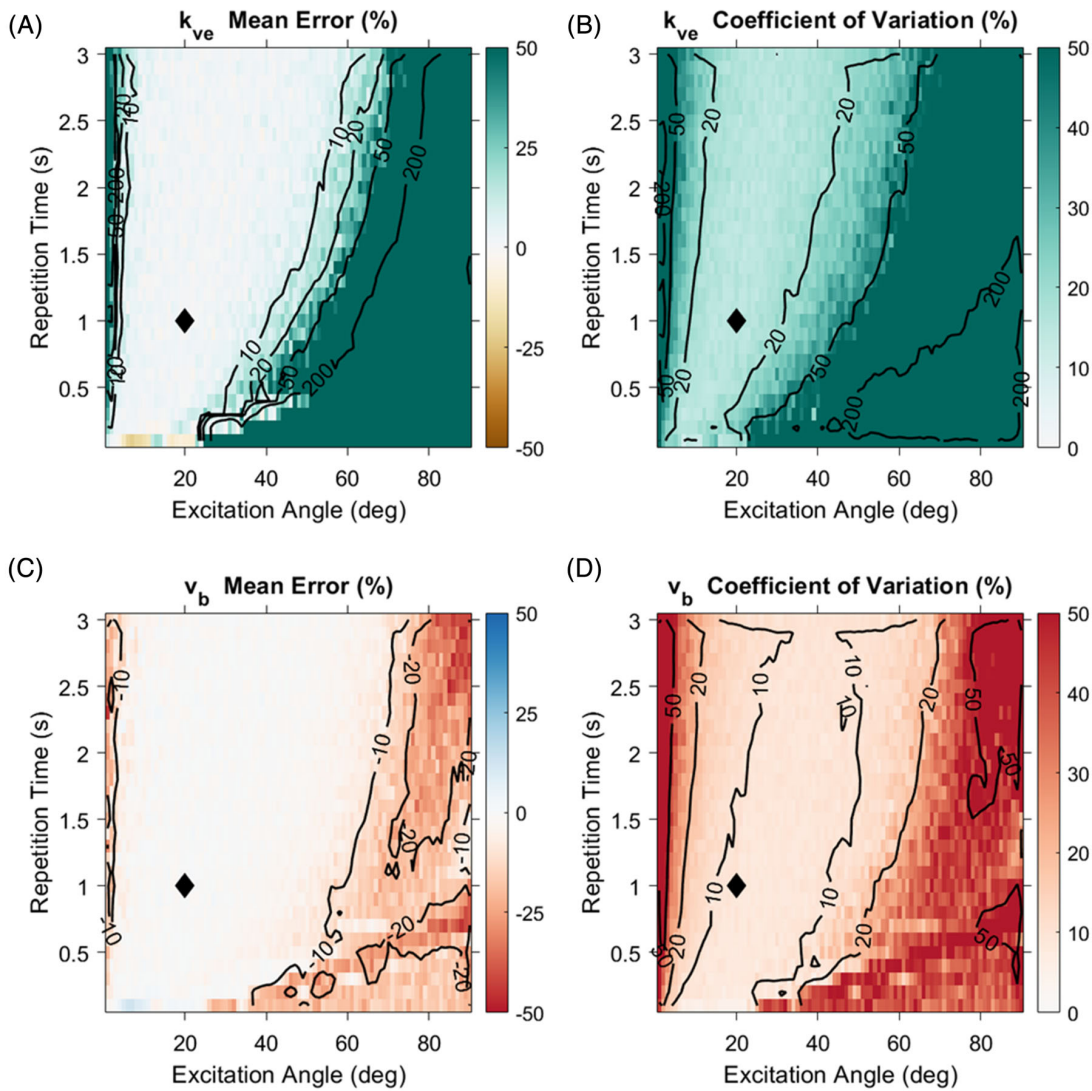
Accuracy and reproducibility for kinetic estimates of blood volume fraction and capillary exchange rate vary with spoiled gradient echo scan parameters. Lighter colors correspond to superior accuracy and reproducibility. Synthetic data were generated using Model I at each set of TR and excitation angle values, and this model was fit to the data after adding zero‐mean Gaussian noise scaled to attain a peak SNR of 25 for TR of 1 s and excitation angle of 20 degrees. Fitting was repeated 100 times with fresh noise at each set of acquisition parameters. Percent mean error (A, C) and coefficient of variation plots (B, D) for the resulting estimates of *k*_*ve*_ (A, B) and *v*_*b*_ (C, D) show a range of acquisition parameters that yield accurate and reproducible estimation of kinetic parameters, with very small (<5 deg) or large (>50 deg) excitation angles generally leading to inferior performance. The black diamond on each plot denotes the standard acquisition parameters for simulations comparing different kinetic models in this work. For clarity, contour lines were calculated from the image data shown after smoothing with a 3x3 mean filter. Results of these acquisition setting simulations for *v*_*ee*_ are shown in Figure S1.

The choice of TR and excitation angle in HP imaging determines the relative amount of agent accumulated in tissue and rate at which non‐renewable magnetization is expunged. Figure 2 depicts the results of fitting Model I to data with variations in these acquisition effects. These data demonstrate that accurate and reproducible estimates of model parameters are obtained with dynamic snapshot imaging at moderate excitation angles of approximately 10 to 40 degrees. At excitations angles outside of this range, overestimation of *k*_*ve*_ and underestimation of *v*_*b*_ occurs with degraded reproducibility of both parameters. Compared to *k*_*ve*_ and *v*_*b*_, the regions of high accuracy and reproducibility for *v*_*ee*_ occur at a narrower range of lower excitation angles (Figure S1). As TR increases, the use of progressively larger excitation angles is feasible, which can be favorable if greater image SNR is needed. Large excitation angles applied at short TR intervals did not permit sufficient extravascular HP agent accumulation for kinetic modeling in these simulations, resulting in very unstable results. At the default acquisition settings used in numerical simulations comparing pharmacokinetic models (1‐s TR, 20‐degree excitation), mean errors for *k*_*ve*_, *v*_*b*_, and *v*_*ee*_ were −1.89%, 1.32%, and 5.91% with coefficients of variation of 14.9%, 8.25%, and 15.6%.

**FIGURE 3:**
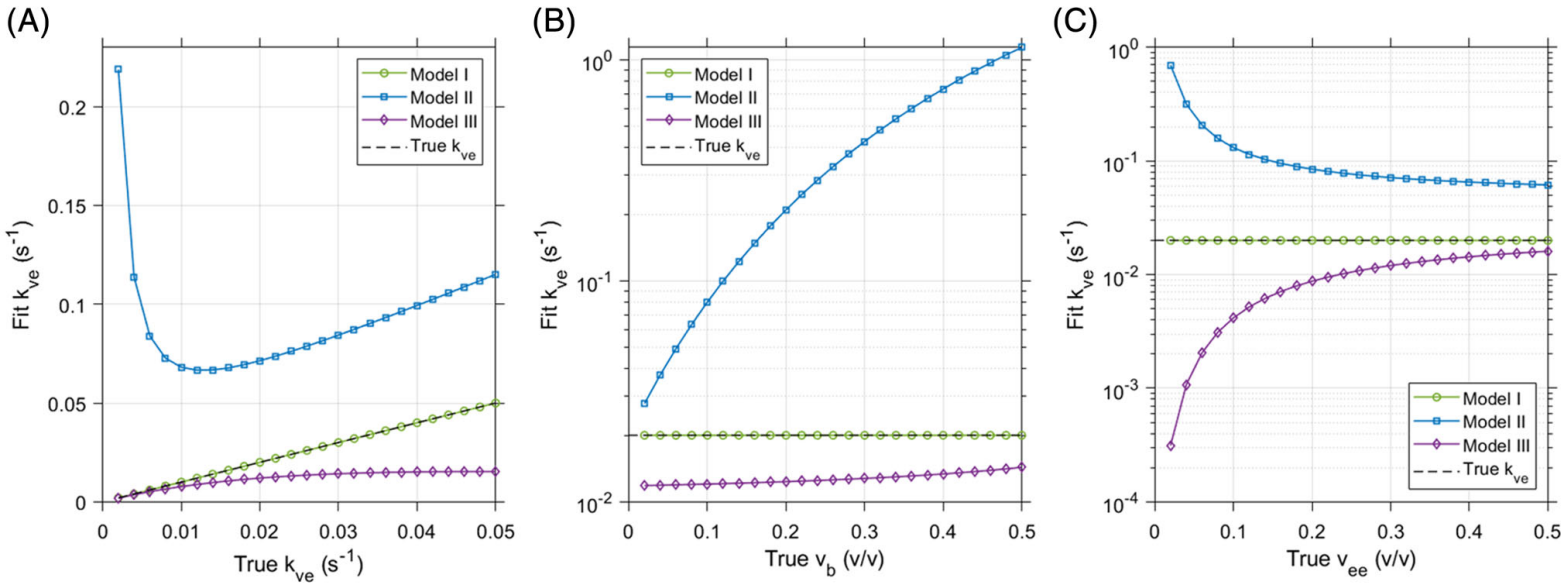
Sensitivity analysis for permeability parameter (*k*_*ve*_) estimation from synthetic data generated with Model I. Noise‐free data were created using a range of parameter values and fit using each model to demonstrate sensitivity of simplified models (II and III) to underlying changes in parameters more fully descriptive of tissue perfusion. (A) As the true *k*_*ve*_ increases, Model II yields nonlinear changes in the estimated *k*_*ve*_ with values consistently greater than the true Model I *k*_*ve*_. In contrast, Model III demonstrates consistent underestimation of *k*_*ve*_. (B) For the conditions simulated with Model I, Model II is highly sensitive to changes in underlying *v*_*b*_ and yields the opposite trend in estimated *k*_*ve*_ values when compared with Model III. (C) When the underlying *v*_*ee*_ increases, both Models II and III provide progressively more accurate *k*_*ve*_ estimates, with Model III demonstrating slightly greater sensitivity to driving *v*_*ee*_. Fitting results from these numerical simulations for *v*_*b*_ and *v*_*e*_ are provided in Figure S2.

The sensitivity of *k*_*ve*_ estimated with Models II and III to underlying changes in each parameter driving Model I are shown in Figure 3. Plots showing the sensitivity of *v*_*b*_ and v_e_ estimates in these simulations are provided in Figure S2. Both simplified models demonstrate nonlinear responses of *k*_*ve*_ estimates to changes in the ground‐truth Model I parameters. Relative to the true Model I, Model II consistently overestimates and Model III consistently underestimates *k*_*ve*_. Models II and III exhibit high sensitivity in *k*_*ve*_ estimates to variation in the volume parameters they do not directly include (*v*_*b*_ and *v*_*ee*_, respectively). Models II and III show opposite trends in *k*_*ve*_ results with changes in true *v*_*b*_, with progressively inaccurate estimation of *k*_*ve*_ for Model II as ground‐truth *v*_*b*_ increases. As the true *v*_*ee*_ increases, *k*_*ve*_ estimates from Models II and III both trend toward more accurate *k*_*ve*_ estimates.

**FIGURE 4:**
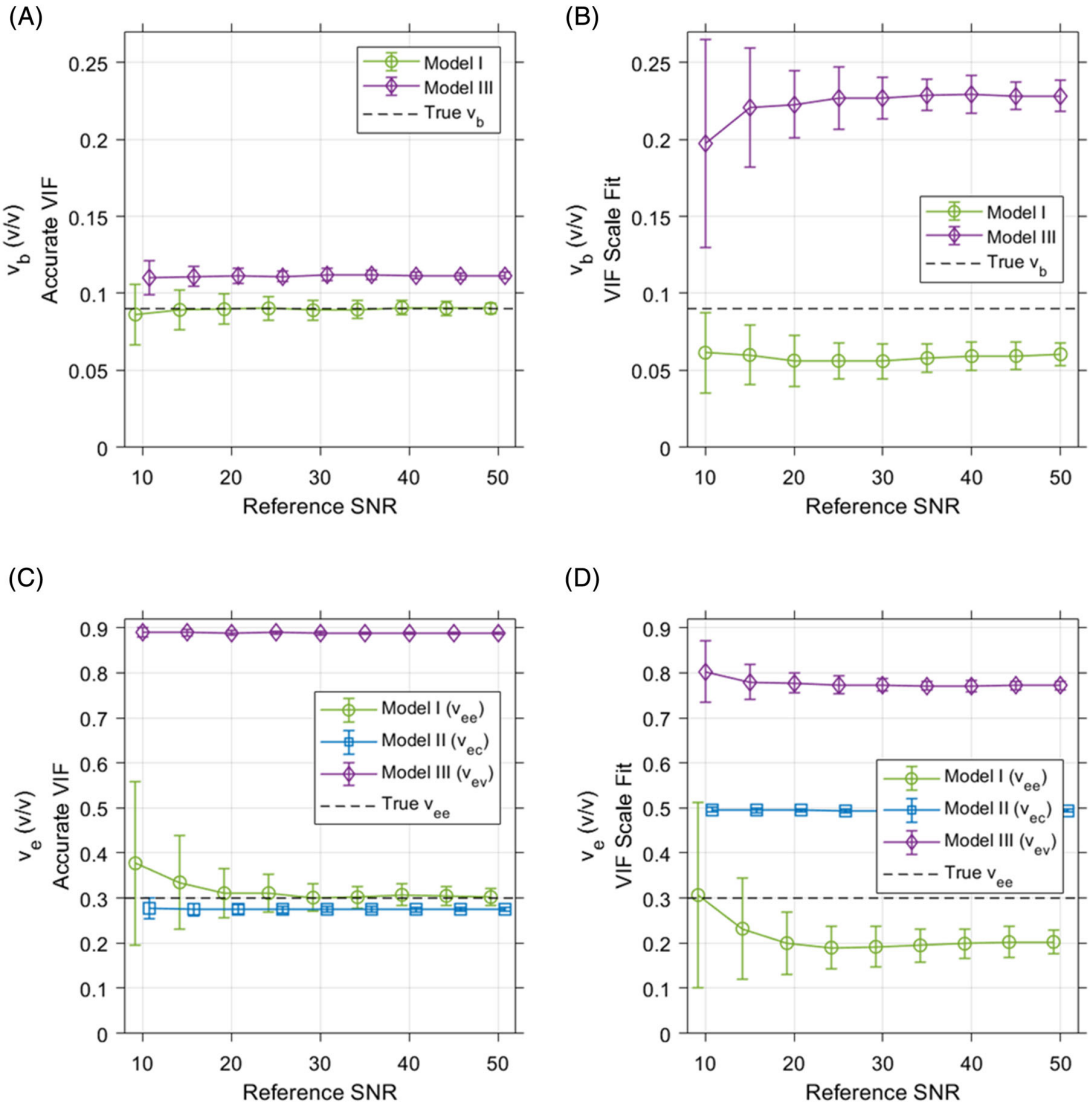
Accuracy and reproducibility for volume parameters estimated from noisy simulated generated with Model I using default values. Plotted points represent the mean fit parameter value at each reference SNR, and error bars depict ±1 SD. (A,C) The simpler Models II and III generally demonstrate greater reproducibility of volume fraction parameters than Model I when the VIF is known accurately, with bias relative to the ground truth values used in generating synthetic data. Compared to the cases where the VIF is known, fitting VIF scale results in added errors and biases for estimation of blood and tissue volume fraction parameters for all three models. (B) Joint fitting of VIF scale and kinetic model parameters results in underestimation of *v*_*b*_ for Model I, and added overestimation bias of *v*_*b*_ for Model III. (D) Additionally, when VIF scale is fit, *v*_*ee*_ is underestimated for Model I and increased *v*_*ec*_ estimates are provided by Model II. Simulations were completed for reference SNRs ranging from 10 to 50 at increments of 5. Where plotted results overlap, the corresponding error bars are offset from one another along the x‐axis solely for visibility. Additional results of these numerical simulations are provided in Figure S3.

**FIGURE 5:**
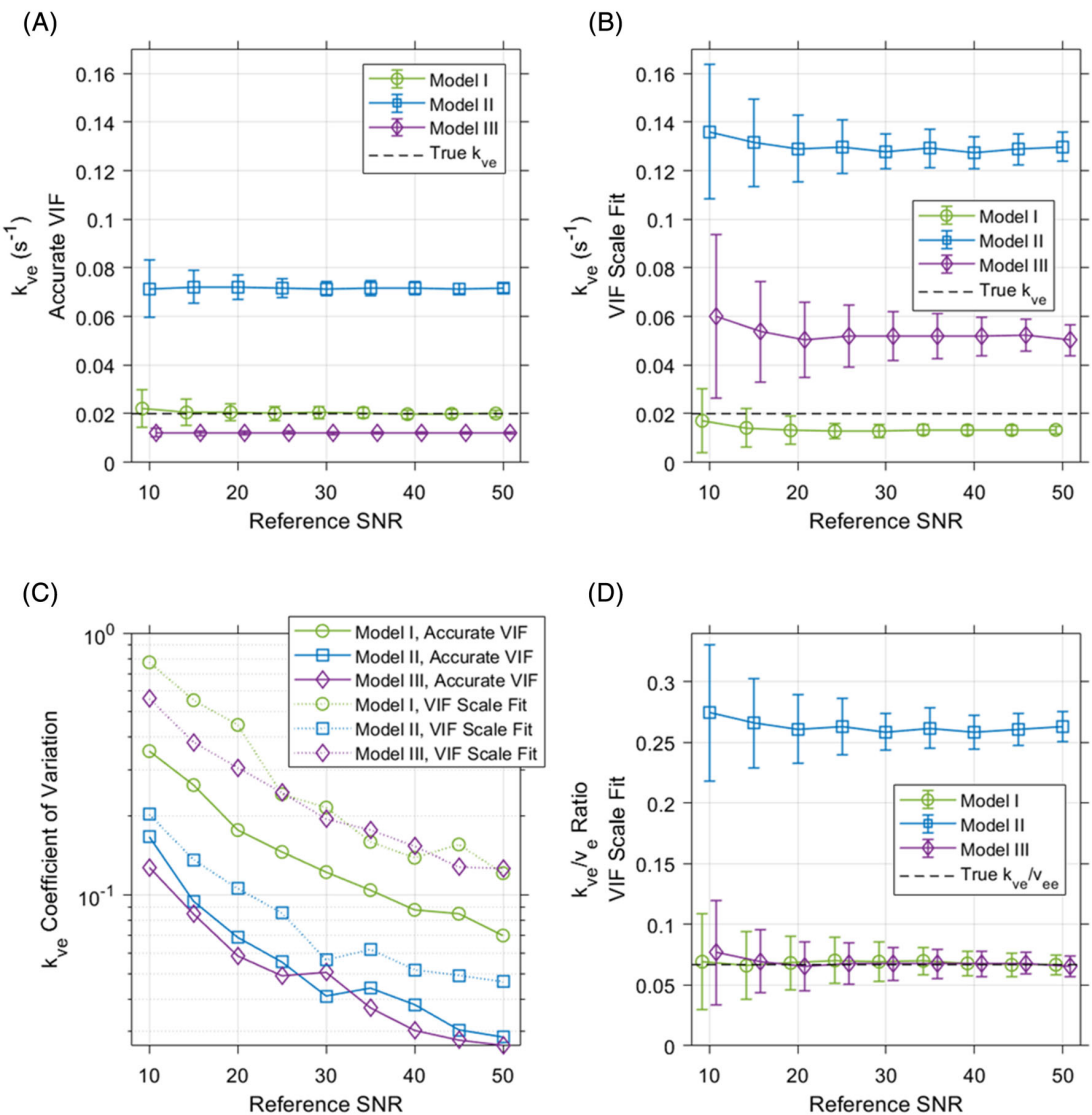
Accuracy and reproducibility for permeability parameters estimated from noisy simulated generated with Model I using default values. Plotted points in A, B, and D represent the mean fit parameter value at each reference SNR, and error bars depict ±1 SD. Compared to results fit with an accurate VIF (A), fitting of VIF scale results in erroneously decreased estimates of *k*_*ve*_ for Model I and a bias toward increased estimates of *k*_*ve*_ for both simplified models (B). (C) The coefficients of variation for *k*_*ve*_ estimates show that Model II provides the most reproducible *k*_*ve*_ results when fitting VIF scale, and also exhibits the least change in coefficient of variation relative to fitting results with an accurate VIF. (D) When VIF scale is fit, the ratio of *k*_*ve*_ to *v*_*e*_ is highly accurate relative to the ground truth value for both Models I and III. Simulations were completed for reference SNRs ranging from 10 to 50 at increments of 5. Where plotted results overlap, the corresponding error bars are offset from one another along the x‐axis solely for visibility. Additional results of these numerical simulations are provided in Figure S3.

The differing complexities of the three kinetic models evaluated in this work result in distinct fitting performances for individual parameters with noisy data. Figure 4 depicts the accuracy and reproducibility of volume fraction parameters fit with each model to noisy data generated with Model I. Compared to Model I results, both Models II and III provide significantly improved reproducibility of volume fraction estimates when fitting noisy data (coefficients of variation for *v*_*b*_ and *v*_*e*_ are provided in Figure S3). Both simplified models also provide superior reproducibility in estimation of *k*_*ve*_ than Model I when models are fit with the true VIF amplitude (Figure 5C). At a reference SNR of 25, coefficients of variation in *k*_*ve*_ for Models I, II, and III in these cases are 14.6%, 5.53%, and 4.9%, respectively.

For each model, noisy data were fit both with an accurate VIF and with the VIF amplitude as an additional fit parameter. Fitting this VIF scaling factor emulates the implementation of these models for image data with non‐uniformities in transmit and/or receive coil sensitivity. In such cases, a VIF measured directly in dynamic imaging must be rescaled to account for spatial variations in coil sensitivity in tissues of interest. When noisy data are fit with joint estimation of VIF amplitude, added bias occurs toward *v*_*b*_ underestimation in Model I and overestimation in Model III, with worse *v*_*b*_ reproducibility in both models relative to the results from fits with an accurate VIF (Figure 4A,B). Reproducibility of *v*_*ev*_ in Model II generally improves when fitting VIF amplitude, but with a strong bias toward increased values relative to the accurate VIF results (Figure 4C,D). The opposite results are seen for *v*_*ee*_ in Model I, with consistent underestimation and degraded reproducibility occurring when VIF scale is fit. All models exhibit worse *k*_*ve*_ reproducibility when VIF amplitude is fit relative to when an accurate VIF is used (Figure 5C). Of the three models used, Model II provides the best reproducibility in *k*_*ve*_ estimation when VIF amplitude is fit, and shows the smallest change in *k*_*ve*_ coefficient of variation relative to fitting with an accurate VIF. Model III demonstrates the largest increase in *k*_*ve*_ coefficient of variation with added estimation of VIF amplitude. Coefficients of variation in *k*_*ve*_ with VIF scale fitting at a reference SNR of 25 for Models I, II, and III are 24.3%, 8.52%, and 24.6%, respectively.

Substantial added bias in *k*_*ve*_ estimation occurs when VIF amplitude is fit (Figure 5B). In this situation, a unique solution for the model parameters being fit does not exist. When compared to results obtained with an accurate VIF, *k*_*ve*_ estimates decrease for Model I and increase for both simplified models. These differences in parameter bias that vary between models result from mis‐estimations of the VIF amplitude (Figure S3C). In practice, scaling the VIF amplitude is equivalent to scaling total tissue signal for all models used in this work. However, the differing compartmentalizations of signals assumed by each model result in disparate effects of VIF amplitude mis‐estimation on tissue parameters. One approach to address these effects is through ratios of model parameter estimates, in which the bias induced in one parameter by error in VIF amplitude is compensated by the respective bias in another parameter. The *k*_*ve*_/*v*_*e*_ ratios resulting from fitting Models I and III with joint estimation of VIF amplitude were highly accurate relative to the ground truth value (Figure 5D). Although this ratio was consistently overestimated for Model II, the coefficients of variation in these ratios show similar reproducibilities to the corresponding *k*_*ve*_ estimates for each model. At a reference SNR of 25, the coefficients of variation in these *k*_*ve*_/*v*_*e*_ ratio values are 26.9%, 8.86%, and 25.4% for Models I–III.

### 3.2 In vivo thyroid tumor imaging

**FIGURE 6:**
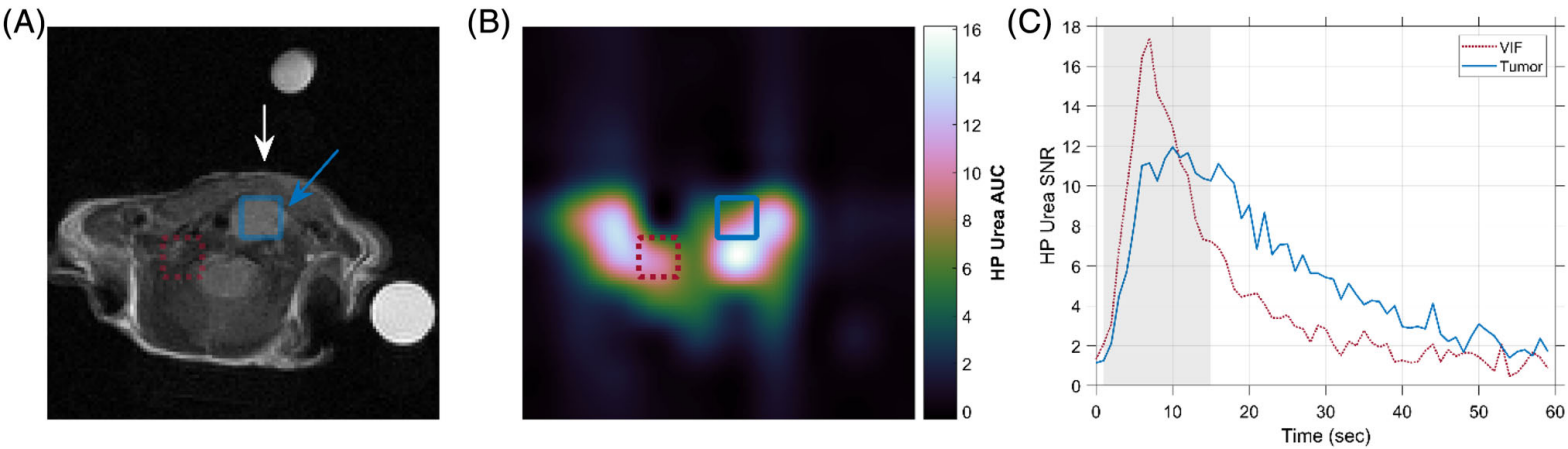
Representative HP urea imaging dataset for a mouse with orthotopic thyroid tumor. (A) An axial T2‐weighted RARE image depicts the thyroid tumor (blue arrow) and approximate position of the ^13^C surface receive coil (white arrow). The area under the curve (AUC) urea image in panel B is averaged over the first 15 s of HP agent signal and interpolated for display to the same resolution as the anatomical image in panel A. The squares overlaid on both images denote the locations of the voxels in the native resolution HP urea images representing VIF (red dashed outline) and tumor (blue outline) signals. The HP urea signal for these VIF and tumor voxels are plotted against time in panel C, with the timepoints averaged in the AUC urea image highlighted in gray.

Illustrative anatomical and HP urea images of a mouse bearing an orthotopic thyroid tumor are shown in Figure 6. Voxel positions representing the VIF shape (red dashed square) and tumor signal (blue square) are highlighted, with the corresponding HP urea signals plotted over time. Intense HP urea signals coincide with the positions of the major blood vessels in the neck, which may contribute to signal within the tumor voxel due to partial volume averaging for the relatively coarse spatial resolution of these HP images (2.5 × 2.5 × 10 mm^3^). These tumor voxel HP urea timecourses nevertheless exhibit signals that are delayed and dispersed in time relative to the VIF voxel time course, consistent with ^13^C urea retention in tumor tissue. The mean peak SNR values for HP signal in tumor voxels was 13, with a range of 7 to 21.

**FIGURE 7:**
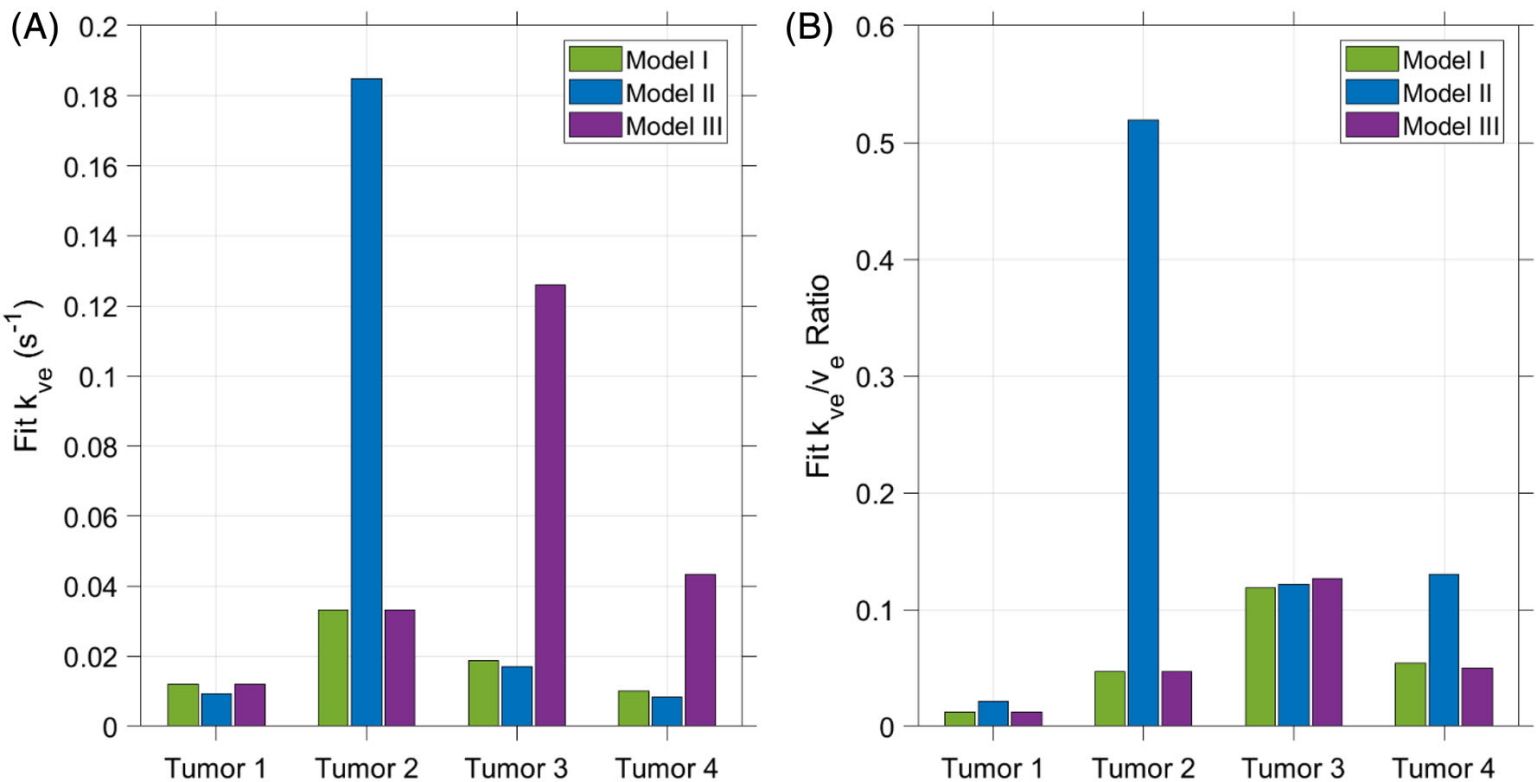
Values for *k*_*ve*_ (A) and *k*_*ve*_/*v*_*e*_ ratio (B) obtained by fitting pharmacokinetic models to HP urea signals in murine orthotopic thyroid tumors with joint estimation of VIF scaling. In two datasets (Tumors 1 and 2), fitting results for Models I and III converged to identical values for all parameters. While the relative magnitudes of fit *k*_*ve*_ values in these in vivo data do not consistently match those noted in simulations comparing models, the *k*_*ve*_/*v*_*e*_ ratios for Models I and III are roughly equal in all cases but may differ for Model II. This corroborates the notable result from simulations that the *k*_*ve*_/*v*_*e*_ ratio can correct for biases introduced in individual parameter estimates when fitting Models I and III (but not Model II) with VIF scaling. The remaining model parameter value estimates for these in vivo data are presented in Figure S4.

Results of fitting pharmacokinetic models to HP urea signal in the four tumors imaged are presented in Figure 7 (*k*_*ve*_ and *k*_*ve*_/*v*_*e*_ ratio) and supplementary Figure S4 (volume fractions and VIF scale factor). While these results do not consistently match every trend demonstrated in our simulations, it is particularly notable that the *k*_*ve*_/*v*_*e*_ ratios representing the estimated rates of HP urea signal decay due to physiological washout demonstrate similar trends as in simulation (Figure 5D). Specifically, this ratio obtained by fitting Models I and III to these in vivo data are roughly equal in all cases, while the corresponding ratio for Model II is greater in most cases.

## 4 DISCUSSION

This work introduces three pharmacokinetic modeling methods for quantification of substrate delivery in dynamic HP urea images and evaluates their performance. Pharmacokinetic modeling entails trade‐offs between model complexity and sensitivity to unmodeled effects. Parameter values obtained by fitting oversimplified models therefore suffer substantial bias when they fail to capture the physiological parameters relevant to signal evolution. However, when comparing experimental groups using a consistent model, the most critical considerations in model selection are the inclusion of appropriate parameters and the reliability of relevant results under experimental conditions. In this context, models such as Models II and III in this work may in some cases be preferred for improved reproducibility of key physiological parameters.

The simulation conditions used throughout this work are based primarily on preclinical imaging results in solid tumors for HP pyruvate, which is of similar molecular size as urea but exhibits different facility of cellular transport within many tissues. Tumor vasculature is highly permeable, and prior studies of HP urea have noted apparent sequestration of signal to blood vessels in normal brain tissue, but greater vascular permeability and tissue distribution in normal liver and solid tumors.^6^ Significant deviations from our assumed default parameter set could affect the relative performance of our models in deriving accurate and reproducible measures of HP signal compartmentalization and vascular permeability. Similarly, significant violations of the simplifying assumptions inherent to Models II and III may yield kinetic modeling results that suffer from poor accuracy and physiological interpretability. Specifically, Model II, as with the Tofts model for DCE‐MRI,^33^ may be expected to provide reliable results for tissues that are not well‐vascularized. Model III, in contrast, might be expected to perform well in tissues through which the HP perfusion agent more freely crosses cellular membranes in the extravascular space. These subjects warrant further study specific to organs and diseases for which these kinetic modeling approaches can hold value, and the modeling framework we provide can be easily adapted to match the appropriate conditions for specific biological targets.

The imaging results for the mice bearing anaplastic thyroid tumors are provided to demonstrate the feasibility of our kinetic modeling strategies in vivo. These results demonstrate the efficacy of *k*_*ve*_/*v*_*e*_ ratios in mitigating the impacts of fitting a VIF scale factor along with kinetic model parameters, which may often be necessary when using ^13^C coils with non‐uniform transmit and/or receive sensitivities. While these in vivo data support our simulation results for *k*_*ve*_/*v*_*e*_ ratios, only limited conclusions may be derived from them due to the small sample size and relatively low SNR of HP urea in tumor voxels. Due to partial volume averaging in the voxels analyzed (both VIF and tumor) and the variance shown in numerical simulations in pharmacokinetic modeling results for data with low reference SNRs, further research is needed before the modeling methods presented here may be considered fully validated.

The quantification methods presented here may not be suitable for modeling HP urea signal evolution in healthy tissues containing capillaries with continuous endothelium restricting extravasation. For cases in which the HP agent is restricted to vascular spaces, modeling methods similar to those used in dynamic susceptibility contrast (DSC) ‐MRI may be more suitable.^34^ In cardiac perfusion imaging, the use of flow‐sensitizing gradients to selectively suppress signals moving within the heart chambers during first‐pass imaging of HP urea can obviate model‐based quantification and permit direct measurement of capillary perfusion.^10^, ^11^ In the context of kidney imaging with HP urea, various alternative analysis methods have been reported. Using time‐resolved SSFP imaging sequences, HP urea transport in the kidney has been evaluated based on spatial signal distributions,^35^, ^36^ measurements of renal cortex perfusion,^9^ and the calculated rate of osmolyte clearance.^37^ Measurement of HP urea T2 relaxation using radial fast spin echo (FSE) sequences has been demonstrated to be a promising biomarker of kidney disease, with preclinical applications reported in diabetic neuropathy,^8^ and acute ischemic kidney injuries.^38^, ^39^ Compared to the snapshot gradient‐echo EPI method we use for proof‐of‐concept imaging of HP urea in the murine kidney, these SSFP and FSE methods benefit from superior spatial resolution but inferior temporal resolution. Although the simulation conditions in this work correspond primarily to HP urea in cancer, our models may also be applicable to dynamic imaging of other HP perfusion agents, with appropriate consideration for the differences in capillary and erythrocyte permeability to such agents.

Assessment of the separate effects of acquisition settings, noise level and varying true pharmacokinetic parameter values through phantom or in vivo experiments is impractical, so these effects were evaluated in numerical simulations. Our results account for many of the practical aspects of these experiments, but are not exhaustive. Confounds such as inaccuracies in VIF timing and shape, and errors in assumed T1 and excitation angle were not simulated and require further study. Additionally, our simulations assume a single, fixed T1 value for all modeled compartments and a VIF shape that is unchanged by the effects of RF signal depletion on HP urea circulating in the vascular system. The blood in the imaged volume is assumed to be fully replenished during the intervals between dynamic imaging timepoints. These assumptions may be violated in vivo depending on the tissues of interest and the fraction of circulating blood exposed to the RF excitation field. Errors in the assumed rates of HP signal depletion due to T1 relaxation and RF excitation can be expected to confound the modeling results of HP agent washout represented by the parameters *k*_*ve*_ and *v*_*e*_ in our modeling methods. It must be noted that all of these effects may differ drastically for distinct organs, RF transmit coil configurations, and pulse sequences. The effects of these factors impacting in vivo HP signal evolution that are not fully accounted for in the modeling summarized in this work will be an area of ongoing study. Both the numerical simulations and in vivo results presented in this work employ snapshot gradient‐echo imaging with a relatively long TR and low excitation angle. Acquisition methods such as balanced SSFP,^12^, ^13^ spin–spin relaxometry,^8^, ^37^, ^39^ and IDEAL^28^ may be used for HP urea imaging, which do not perfectly match the simulated acquisition. The models presented in this work may require modification or be entirely unsuitable for use with imaging strategies that differ significantly from dynamic snapshot imaging.

The choice of a specific method for analysis of HP urea images should carefully consider the reliability of salient results. Our results demonstrate the trade‐offs inherent in model complexity, and will be useful for the selection of optimal analysis methods in HP perfusion imaging.

## Supporting information


**Figure S1.** Accuracy and reproducibility for kinetic estimates of extravascular/extracellular space volume fraction (*v*_*ee*_) vary with spoiled gradient echo scan parameters. Lighter colors correspond to superior accuracy and reproducibility. Synthetic data were generated using Model I at each set of TR and excitation angle values, and this model was fit to the data after adding zero‐mean Gaussian noise scaled to attain a peak SNR of 25 for TR of 1 second and excitation angle of 20 degrees. Fitting was repeated 100 times with fresh noise at each set of acquisition parameters. Percent mean error (**A**) and coefficient of variation (**B**) for the resulting *v*_*ee*_ estimates show a range of acquisition parameters that yield accurate and reproducible estimation of kinetic parameters. The black diamond on each plot denotes the standard acquisition parameters for simulations comparing different kinetic models in this work. For clarity, contour lines were calculated from the image data shown after smoothing with a 3x3 mean filter.
**Figure S2.** Sensitivity analysis for volume fraction parameter estimations, derived by fitting all models to noise‐free synthetic data generated with Model I. Similar sensitivity analysis results for kve are shown in Figure 3. Model III maps changes in kve strongly as changes in vb. For incremental changes in driving vb, linear responses are seen in both volume parameter estimates for Model III. Models II and III show generally opposing trends in ve estimates with changes in driving vb. Responses of volume fraction estimates to changes in true vee are non‐linear for both simplified models.
**Figure S3.** Coefficient of variation plots for vb and ve depict the relative reproducibility of volume fraction parameter estimates for each model fit to noisy data both with an accurate VIF and with joint estimation of VIF amplitude (A, B). Both simplified Models II and III demonstrate improved reproducibility of volume parameters relative to Model I. All models demonstrate less reproducible estimation of volume fractions when VIF amplitude is fit, except for Model II (B). Subfigure C depicts the accuracy and reproducibility of the VIF scale factor fitting results, with points representing the mean fit VIF scale factor at each reference SNR, and error bars denoting ±1 standard deviation. Both Models I and II exhibit wide variability in VIF scale estimates at very low SNRs, in contrast with Model II which provides very consistent and reproducible VIF amplitude estimates across all SNRs tested. For reference SNRs greater than 10, Model I consistently overestimates VIF scale and both Models II and III underestimate it. When compared with data that were fit with an accurate VIF, these mis‐estimations of the VIF amplitude result in added bias for pharmacokinetic parameter results across all models (Figures 4 and 5).
**Figure S4.** Pharmacokinetic modeling results for volume fraction parameters and VIF scale factors fit to HP urea signals in murine orthotopic thyroid tumors. In two datasets (Tumors 1 and 2), fitting results for Models I and III converge to identical values for all parameters. While the relative magnitudes of individual parameter estimates fit to our three models do not always match the trends observed in simulations, these in vivo data support the notable result from simulations that the *k*_*ve*_/*v*_*e*_ ratio can correct for biases introduced in individual parameter estimates when fitting VIF scale factor for Models I and III (but not Model II). The *k*_*ve*_ and *k*_*ve*_/*v*_*e*_ ratio value estimates from these kinetic model fits to in vivo data are presented in Figure 7.

## Data Availability

MATLAB code used for simulation and modeling in this work is publicly available online (https://github.com/mda‐mrsl/HP‐Urea‐PK/commit/0be5139bbe0269fd383a7206d4dfe4aa31e3f505).

## References

[1] Wang ZJ , Ohliger MA , Larson PEZ , et al. Hyperpolarized 13C MRI: state of the art and future directions. Radiology. 2019;291:273‐284. doi:10.1148/radiol.2019182391 30835184 PMC6490043

[2] Kurhanewicz J , Vigneron DB , Ardenkjaer‐Larsen JH , et al. Hyperpolarized 13C MRI: path to clinical translation in oncology. Neoplasia. 2019;21:1‐16. doi:10.1016/j.neo.2018.09.006 30472500 PMC6260457

[3] Bankson JA , Walker CM , Ramirez MS , et al. Kinetic modeling and constrained reconstruction of hyperpolarized [1‐13C]‐pyruvate offers improved metabolic imaging of tumors. Cancer Res. 2015;75:4708‐4717. doi:10.1158/0008-5472.CAN-15-0171 26420214 PMC4651725

[4] Golman K , Ardenkjaer‐Larsen JH , Petersson JS , Mansson S , Leunbach I . Molecular imaging with endogenous substances. Proc Natl Acad Sci U S A. 2003;100:10435‐10439. doi:10.1073/pnas.1733836100 12930896 PMC193579

[5] Svensson J , Månsson S , Johansson E , Petersson JS , Olsson LE . Hyperpolarized 13C MR angiography using trueFISP. Magn Reson Med. 2003;50:256‐262. doi:10.1002/mrm.10530 12876701

[6] von Morze C , Bok RA , Reed GD , Ardenkjaer‐Larsen JH , Kurhanewicz J , Vigneron DB . Simultaneous multiagent hyperpolarized (13)C perfusion imaging. Magn Reson Med. 2014;72:1599‐1609. doi:10.1002/mrm.25071 24382698 PMC4077988

[7] Reeder RF , Harbaugh RE . Administration of intravenous urea and normal saline for the treatment of hyponatremia in neurosurgical patients. J Neurosurg. 1989;70:201‐206. doi:10.3171/jns.1989.70.2.0201 2913218

[8] Laustsen C , Stokholm Nørlinger T , Christoffer Hansen D , et al. Hyperpolarized 13C urea relaxation mechanism reveals renal changes in diabetic nephropathy. Magn Reson Med. 2016;75:515‐518. doi:10.1002/mrm.26036 26584247 PMC4738460

[9] Nielsen P , Hansen ESS , Nørlinger T , et al. Renal ischemia and reperfusion assessment with three‐dimensional hyperpolarized 13C,15N2‐urea. Magn Reson Med. 2016;76: 1524–1530. doi:10.1002/mrm.26377 27548739

[10] Lau AZ , Miller JJ , Robson MD , Tyler DJ . Cardiac perfusion imaging using hyperpolarized (13)C urea using flow sensitizing gradients. Magn Reson Med. 2016;75:1474‐1483. doi:10.1002/mrm.25713 25991580 PMC4556069

[11] Lau AZ , Miller JJ , Robson MD , Tyler DJ . Simultaneous assessment of cardiac metabolism and perfusion using copolarized [1‐13 C]pyruvate and 13 C‐urea. Magn Reson Med. 2017;77:151‐158. doi:10.1002/mrm.26106 26743440 PMC5217077

[12] Qin H , Tang S , Riselli AM , et al. Clinical translation of hyperpolarized 13 C pyruvate and urea MRI for simultaneous metabolic and perfusion imaging. Magn Reson Med. 2022;87:138‐149. doi:10.1002/mrm.28965 34374471 PMC8616838

[13] Liu X , Tang S , Mu C , et al. Development of specialized magnetic resonance acquisition techniques for human hyperpolarized [13 C,15 N2 ]urea + [1‐13 C]pyruvate simultaneous perfusion and metabolic imaging. Magn Reson Med. 2022;88:1039‐1054. doi:10.1002/mrm.29266 35526263 PMC9810116

[14] Wilson DM , Keshari KR , Larson PEZ , et al. Multi‐compound polarization by DNP allows simultaneous assessment of multiple enzymatic activities in vivo. J Magn Reson. 2010;205:141‐147. doi:10.1016/j.jmr.2010.04.012 20478721 PMC2885774

[15] Chen HY , Larson PEZ , Bok RA , et al. Assessing prostate cancer aggressiveness with hyperpolarized dual‐agent 3D dynamic imaging of metabolism and perfusion. Cancer Res. 2017;77:3207‐3216. doi:10.1158/0008-5472.CAN-16-2083 28428273 PMC5484421

[16] Qin H , Zhang V , Bok RA , et al. Simultaneous metabolic and perfusion imaging using hyperpolarized 13C MRI can evaluate early and dose‐dependent response to radiation therapy in a prostate cancer mouse model. Int J Radiat Oncol Biol Phys. 2020;107:887‐896. doi:10.1016/j.ijrobp.2020.04.022 32339646 PMC7381368

[17] Lee JE , Diederich CJ , Bok R , et al. Assessing high‐intensity focused ultrasound treatment of prostate cancer with hyperpolarized 13 C dual‐agent imaging of metabolism and perfusion. NMR Biomed. 2019;32:e3962. doi:10.1002/nbm.3962 30022550 PMC6338537

[18] Sun CY , Walker CM , Michel KA , Venkatesan AM , Lai SY , Bankson JA . Influence of parameter accuracy on pharmacokinetic analysis of hyperpolarized pyruvate. Magn Reson Med. 2018;79:3239‐3248. doi:10.1002/mrm.26992 29090487 PMC5843516

[19] Larson PEZ , Chen HY , Gordon JW , et al. Investigation of analysis methods for hyperpolarized 13C‐pyruvate metabolic MRI in prostate cancer patients. NMR Biomed. 2018;31:e3997. doi:10.1002/nbm.3997 30230646 PMC6392436

[20] Tofts PS . Modeling tracer kinetics in dynamic Gd‐DTPA MR imaging. J Magn Reson Imaging. 1997;7:91‐101. doi:10.1002/jmri.1880070113 9039598

[21] Sands JM . Molecular mechanisms of urea transport. J Membr Biol. 2003;191:149‐163. doi:10.1007/s00232-002-1053-1 12571750

[22] Pagès G , Puckeridge M , Liangfeng G , et al. Transmembrane exchange of hyperpolarized 13C‐urea in human erythrocytes: subminute timescale kinetic analysis. Biophys J. 2013;105:1956‐1966. doi:10.1007/s00232-002-1053-1 24209840 PMC3824547

[23] Tofts PS , Kermode AG . Measurement of the blood‐brain barrier permeability and leakage space using dynamic MR imaging. 1. Fundamental concepts. Magn Reson Med. 1991;17:357‐367. doi:10.1002/mrm.1910170208 2062210

[24] Walker CM , Chen Y , Lai SY , Bankson JA . A novel perfused Bloch‐McConnell simulator for analyzing the accuracy of dynamic hyperpolarized MRS. Med Phys. 2016;43:854‐864. doi:10.1118/1.4939877 26843246 PMC4723412

[25] Milshteyn E , Reed GD , Gordon JW , et al. Simultaneous T1 and T2 mapping of hyperpolarized 13C compounds using the bSSFP sequence. J Magn Reson. 2020;312:106691. doi:10.1016/j.jmr.2020.106691 32058912 PMC7227792

[26] Kazan SM , Reynolds S , Kennerley A , et al. Kinetic modeling of hyperpolarized (13)C pyruvate metabolism in tumors using a measured arterial input function. Magn Reson Med. 2013;70:943‐953. doi:10.1002/mrm.24546 23169010

[27] Walker CM , Fuentes D , Larson PEZ , Kundra V , Vigneron DB , Bankson JA . Effects of excitation angle strategy on quantitative analysis of hyperpolarized pyruvate. Magn Reson Med. 2019;81:3754‐3762. doi:10.1002/mrm.27687 30793791 PMC6435389

[28] Michel KA , Ragavan M , Walker CM , Merritt ME , Lai SY , Bankson JA . Comparison of selective excitation and multi‐echo chemical shift encoding for imaging of hyperpolarized [1‐13C]pyruvate. J Magn Reson. 2021;325:106927. doi:10.1016/j.jmr.2021.106927 33607386 PMC8009829

[29] Walker CM , Gordon JW , Xu Z , et al. Slice profile effects on quantitative analysis of hyperpolarized pyruvate. NMR Biomed. 2020;33:e4373. doi:10.1002/nbm.4373 32743881 PMC7484340

[30] Coleman TF , Li Y . An interior trust region approach for nonlinear minimization subject to bounds. SIAM J Optim. 1996;6:418‐445. doi:10.1137/0806023

[31] Sandulache VC , Skinner HD , Wang Y , et al. Glycolytic inhibition alters anaplastic thyroid carcinoma tumor metabolism and improves response to conventional chemotherapy and radiation. Mol Cancer Ther. 2012;11:1373‐1380. doi:10.1158/1535-7163.MCT-12-0041 22572813 PMC3856684

[32] Marco‐Rius I , Gordon JW , Mattis AN , et al. Diffusion‐weighted imaging of hyperpolarized [13 C]urea in mouse liver. J Magn Reson Imaging. 2018;47:141‐151. doi:10.1002/jmri.25721 28419644 PMC5645231

[33] Sourbron SP , Buckley DL . On the scope and interpretation of the Tofts models for DCE‐MRI. Magn Reson Med. 2011;66:735‐745. doi:10.1002/mrm.22861 21384424

[34] von Morze C , Larson PEZ , Hu S , et al. Imaging of blood flow using hyperpolarized [(13)C]urea in preclinical cancer models. J Magn Reson Imaging. 2011;33:692‐697. doi:10.1002/jmri.22484 21563254 PMC3566235

[35] von Morze C , Bok RA , Sands JM , Kurhanewicz J , Vigneron DB . Monitoring urea transport in rat kidney in vivo using hyperpolarized 13C magnetic resonance imaging. Am J Physiol‐Renal Physiol. 2012;302:F1658‐F1662. doi:10.1152/ajprenal.00640.2011 22492940 PMC3378100

[36] Hansen ESS , Stewart NJ , Wild JM , Stødkilde‐Jørgensen H , Laustsen C . Hyperpolarized 13 C,15 N2 ‐urea MRI for assessment of the urea gradient in the porcine kidney. Magn Reson Med. 2016;76:1895‐1899. doi:10.1002/mrm.26483 27670826

[37] Mariager CØ , Nielsen PM , Qi H , Schroeder M , Bertelsen LB , Laustsen C . Can hyperpolarized 13C‐urea Be used to assess glomerular filtration rate? A retrospective study. Tomography. 2017;3:146‐152. doi:10.18383/j.tom.2017.00010 30042978 PMC6024438

[38] Mariager CØ , Nielsen PM , Qi H , Ringgaard S , Laustsen C . Hyperpolarized 13 C,15 N2 ‐urea T2 relaxation changes in acute kidney injury. Magn Reson Med. 2018;80:696‐702. doi:10.1002/mrm.27050 29285782

[39] Grist JT , Mariager CØ , Qi H , Nielsen PM , Laustsen C . Detection of acute kidney injury with hyperpolarized [13 C, 15 N]urea and multiexponential relaxation modeling. Magn Reson Med. 2020;84:943‐949. doi:10.1002/mrm.28134 31840294

