# Supplementary material for: Pharmacokinetic modeling strategies for dynamic hyperpolarized urea imaging
Source: Magn Reson Med. 2025 Oct 12;95(3):1671–83. doi: 10.1002/mrm.70117 (PMC12746374; doi:10.1002/mrm.70117)
Supplement: Supplementary file 1 — Figure S1. Accuracy and reproducibility for kinetic estimates of extravascular/extracellular space volume fraction (v ee ) vary with spoiled gradient echo scan parameters. Lighter colors correspond to superior accuracy and reproducibility. Synthetic data were generated using Model I at each set of TR and excitation angle values, and this model was fit to the data after adding zero‐mean Gaussian noise scaled to attain a peak SNR of 25 for TR of 1 second and excitation angle of 20 degrees. Fitting was repeated 100 times with fresh noise at each set of acquisition parameters. Percent mean error (A) and coefficient of variation (B) for the resulting v ee estimates show a range of acquisition parameters that yield accurate and reproducible estimation of kinetic parameters. The black diamond on each plot denotes the standard acquisition parameters for simulations comparing different kinetic models in this work. For clarity, contour lines were calculated from the image data shown after smoothing with a 3x3 mean filter. Figure S2. Sensitivity analysis for volume fraction parameter estimations, derived by fitting all models to noise‐free synthetic data generated with Model I. Similar sensitivity analysis results for kve are shown in Figure 3. Model III maps changes in kve strongly as changes in vb. For incremental changes in driving vb, linear responses are seen in both volume parameter estimates for Model III. Models II and III show generally opposing trends in ve estimates with changes in driving vb. Responses of volume fraction estimates to changes in true vee are non‐linear for both simplified models. Figure S3. Coefficient of variation plots for vb and ve depict the relative reproducibility of volume fraction parameter estimates for each model fit to noisy data both with an accurate VIF and with joint estimation of VIF amplitude (A, B). Both simplified Models II and III demonstrate improved reproducibility of volume parameters relative to Model I. All models demonstrate [file MRM-95-1671-s001.pdf]

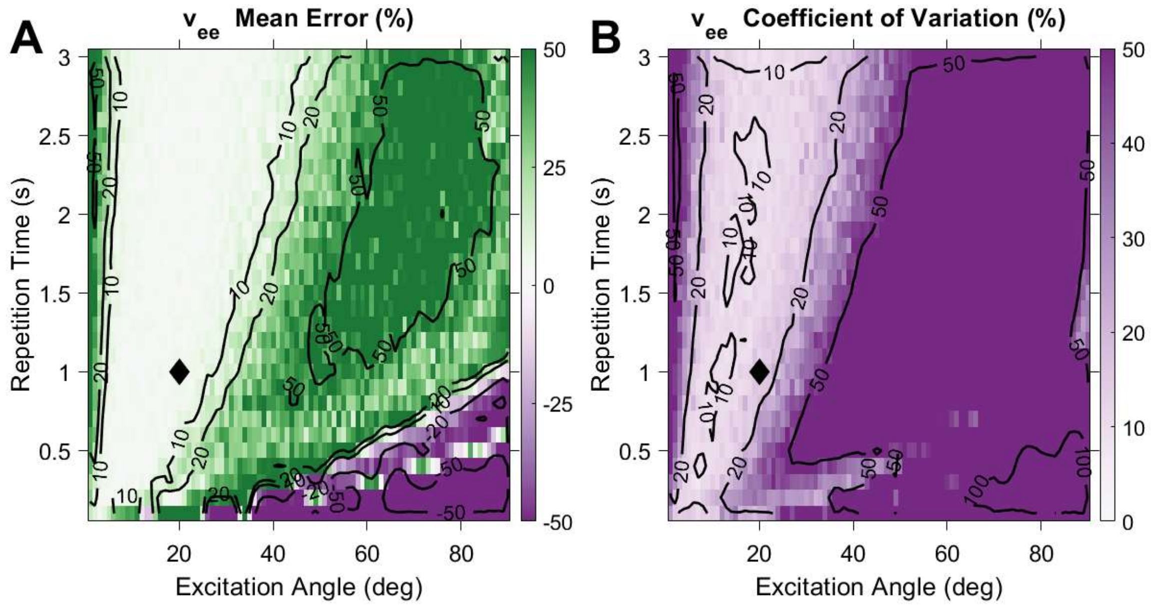

Figure S1. Accuracy and reproducibility for kinetic estimates of extravascular/extracellular space volume fraction (v_{ee}) vary with spoiled gradient echo scan parameters. Lighter colors correspond to superior accuracy and reproducibility. Synthetic data were generated using Model I at each set of TR and excitation angle values, and this model was fit to the data after adding zero-mean Gaussian noise scaled to attain a peak SNR of 25 for TR of 1 second and excitation angle of 20 degrees. Fitting was repeated 100 times with fresh noise at each set of acquisition parameters. Percent mean error (**A**) and coefficient of variation (**B**) for the resulting v_{ee} estimates show a range of acquisition parameters that yield accurate and reproducible estimation of kinetic parameters. The black diamond on each plot denotes the standard acquisition parameters for simulations comparing different kinetic models in this work. For clarity, contour lines were calculated from the image data shown after smoothing with a 3x3 mean filter.

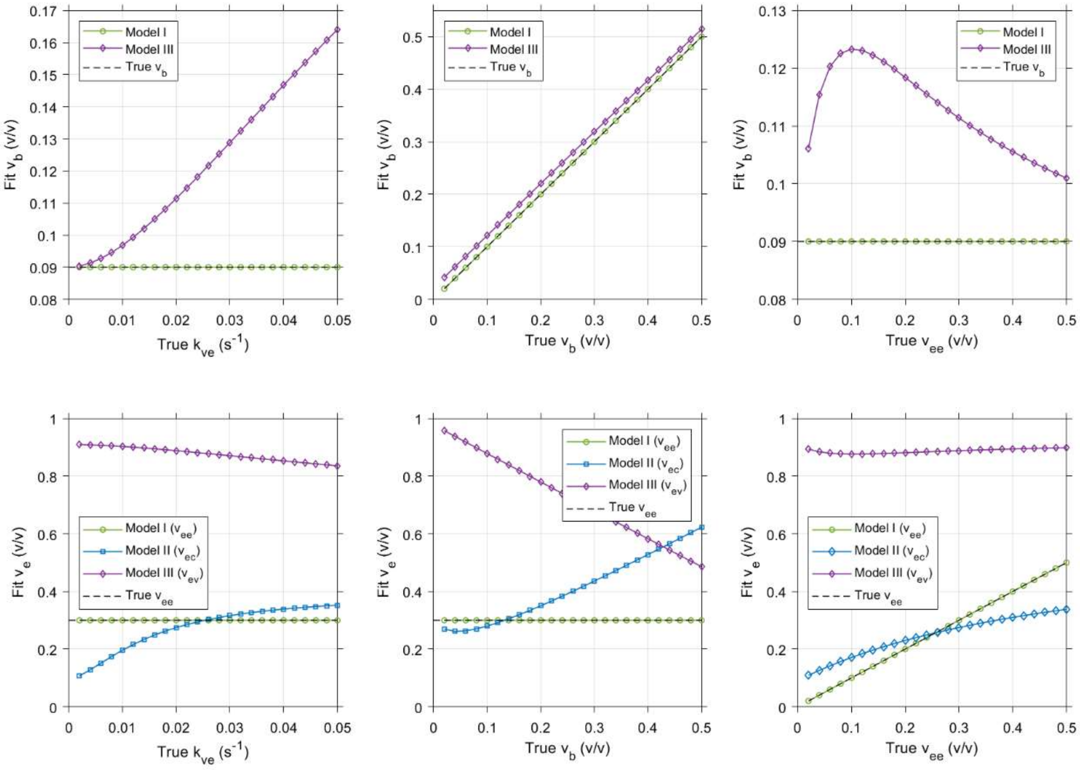

Figure S2. Sensitivity analysis for volume fraction parameter estimations, derived by fitting all models to noise-free synthetic data generated with Model I. Similar sensitivity analysis results for k_{ve} are shown in Figure 3. Model III maps changes in k_{ve} strongly as changes in v_b. For incremental changes in driving v_b, linear responses are seen in both volume parameter estimates for Model III. Models II and III show generally opposing trends in v_e estimates with changes in driving v_b. Responses of volume fraction estimates to changes in true v_{ee} are non-linear for both simplified models.

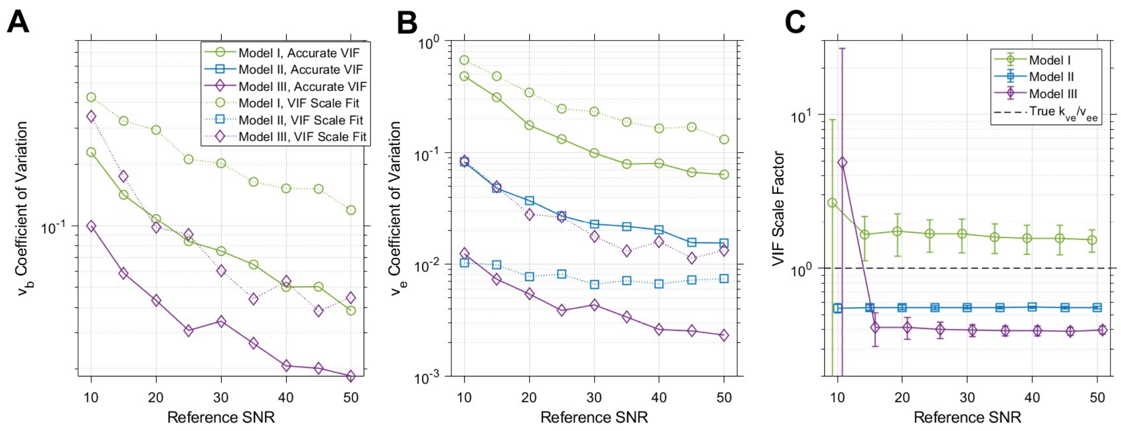

Figure S3. Coefficient of variation plots for v_b and v_e depict the relative reproducibility of volume fraction parameter estimates for each model fit to noisy data both with an accurate VIF and with joint estimation of VIF amplitude (**A**,**B**). Both simplified Models II and III demonstrate improved reproducibility of volume parameters relative to Model I. All models demonstrate less reproducible estimation of volume fractions when VIF amplitude is fit, except for Model II (**B**). Subfigure **C** depicts the accuracy and reproducibility of the VIF scale factor fitting results, with points representing the mean fit VIF scale factor at each reference SNR, and error bars denoting ±1 standard deviation. Both Models I and II exhibit wide variability in VIF scale estimates at very low SNRs, in contrast with Model II which provides very consistent and reproducible VIF amplitude estimates across all SNRs tested. For reference SNRs greater than 10, Model I consistently overestimates VIF scale and both Models II and III underestimate it. When compared with data that were fit with an accurate VIF, these mis-estimations of the VIF amplitude result in added bias for pharmacokinetic parameter results across all models (Figures 4 and 5).

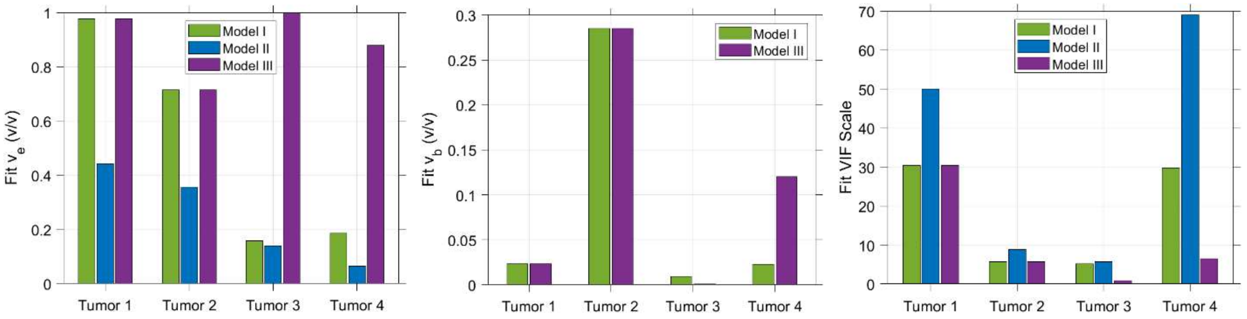

Figure S4. Pharmacokinetic modeling results for volume fraction parameters and VIF scale factors fit to HP urea signals in murine orthotopic thyroid tumors. In two datasets (Tumors 1 and 2), fitting results for Models I and III converge to identical values for all parameters. While the relative magnitudes of individual parameter estimates fit to our three models do not always match the trends observed in simulations, these in vivo data support the notable result from simulations that the k_{ve}/v_e ratio can correct for biases introduced in individual parameter estimates when fitting VIF scale factor for Models I and III (but not Model II). The k_{ve} and k_{ve}/v_e ratio value estimates from these kinetic model fits to in vivo data are presented in Figure 7.
